# Hypervascular gastrointestinal stromal tumor: a rare case report

**DOI:** 10.1097/MS9.0000000000002176

**Published:** 2024-05-15

**Authors:** Firas Khana, Abdulrahman Katerji, Taher Sawas, Jack Zambakjian, Dima Kafa, Abdulmonem Abdullah, Maen Moussa, Nour Lbabidi, Zakaria Al-Najjar, Ahmad Ghazal

**Affiliations:** Departments ofa Medical Imaging and Diagnostic Radiology; b Gastroenterology; c General Surgery, Faculty of Medicine, University of Aleppo, Aleppo University Hospital (AUH), Aleppo, Syria

**Keywords:** case report, computed tomography angiography, gastrointestinal hemorrhage, gastrointestinal stromal tumors, ileum

## Abstract

**Introduction and importance:**

To document a case of an ileal gastrointestinal stromal tumor that caused a massive lower gastrointestinal hemorrhage.

**Case presentation:**

A 55-year-old man presented with multiple episodes of melena and decreased hemoglobin levels. Computed tomography angiography (CTA) revealed a hypervascularized ileal mass.

**Clinical discussion:**

The mass was surgically excised, and the patient’s hemoglobin levels stabilized. Histopathological findings confirmed it to be a low-grade gastrointestinal stromal tumor (G1 GIST).

**Conclusion:**

GISTs are infrequent clinical entities that should be kept in mind when managing patients with gastrointestinal hemorrhage of unknown origin. Using proper imaging modalities is essential for the accurate diagnosis of such tumors, with CTA proving to be particularly effective in identifying hypervascularized tumors.

## Introduction and importance

Highlights

- Gastrointestinal stromal tumors (GISTs) are most commonly found in the stomach and small intestines.
- GISTs may present with gastrointestinal hemorrhage.
- Computed tomography angiography is crucial for identifying hypervascular GISTs.
- Emergent surgery may be necessary in critical GIST scenarios.
- Meticulous postoperative care is essential to ensure a positive response to treatment.

Gastrointestinal stromal tumors (GISTs) are widely recognized as the most common mesenchymal tumors of the gastrointestinal tract, comprising ~0.1–3% of all gastrointestinal tumors^1^.

Typically, patients present with symptoms such as gastrointestinal bleeding and abdominal pain. Additionally, some may experience dyspepsia and early satiety. Interestingly, they are often discovered incidentally on imaging studies or intraoperatively^1,2^.

The objective of this manuscript is to document a rare case of a gastrointestinal stromal tumor occurring in the terminal ileum, and causing massive gastrointestinal hemorrhage.

This case report has been reported in line with the SCARE 2023 criteria^3^.

## Case presentation

A 55-year-old male presented to the emergency department with a 2-day history of dizziness and multiple episodes of melena. The patient had no clinical history and did not report any abdominal pain. There was no drug or allergy history. However, it was noted that the patient was a moderate smoker with a smoking history of 20 pack-years.

Upon initial examination, the patient’s blood pressure was within normal limits, measuring 110/72 mmHg, with an elevated pulse of 110 bpm. Electrocardiography revealed sinus tachycardia with ST-segment depression on leads I, aVL, V5 and V6. His temperature was recorded at 37.2°C, and his oxygen saturation (SpO_2_) on room air at 98%.

Further examination did not reveal any significant abdominal findings. There were no signs of tenderness, guarding or rigidity. Notably, a digital rectal examination confirmed the presence of melena and fresh blood, alongside the identification of an anal fissure.

Initial laboratory investigations revealed a hemoglobin level of 5.6 g/dl, a white blood cell count of 8.8×10^9^/l, a platelet count of 223×10^9^/l, and an international normalized ratio of 1.3. Liver function tests and other biochemical markers yielded normal results.

The patient received resuscitation measures in the emergency department, including intravenous fluids and whole blood transfusions. The patient was then promptly transferred to the ICU and immediately started on omeprazole infusions. Despite initial interventions, the patient experienced hematochezia while maintaining normal blood pressure, although tachycardia persisted with measurements of 120 bpm despite subsequent transfusions.

An urgent esophagogastroduedenoscopy was performed in the ICU and again yielded no significant findings, except for the mucosa appearing pale. Hemoglobin levels remained persistently low at 5.4 g/dl despite giving a transfusion of 5 units of blood.

Due to inconclusive findings from the previous esophagogastroduedenoscopies, a prompt colonoscopy was performed without bowel preparation in an attempt to identify the source of the bleed. Unfortunately, this procedure also failed to pinpoint the exact location of the bleeding.

**Figure 1:**
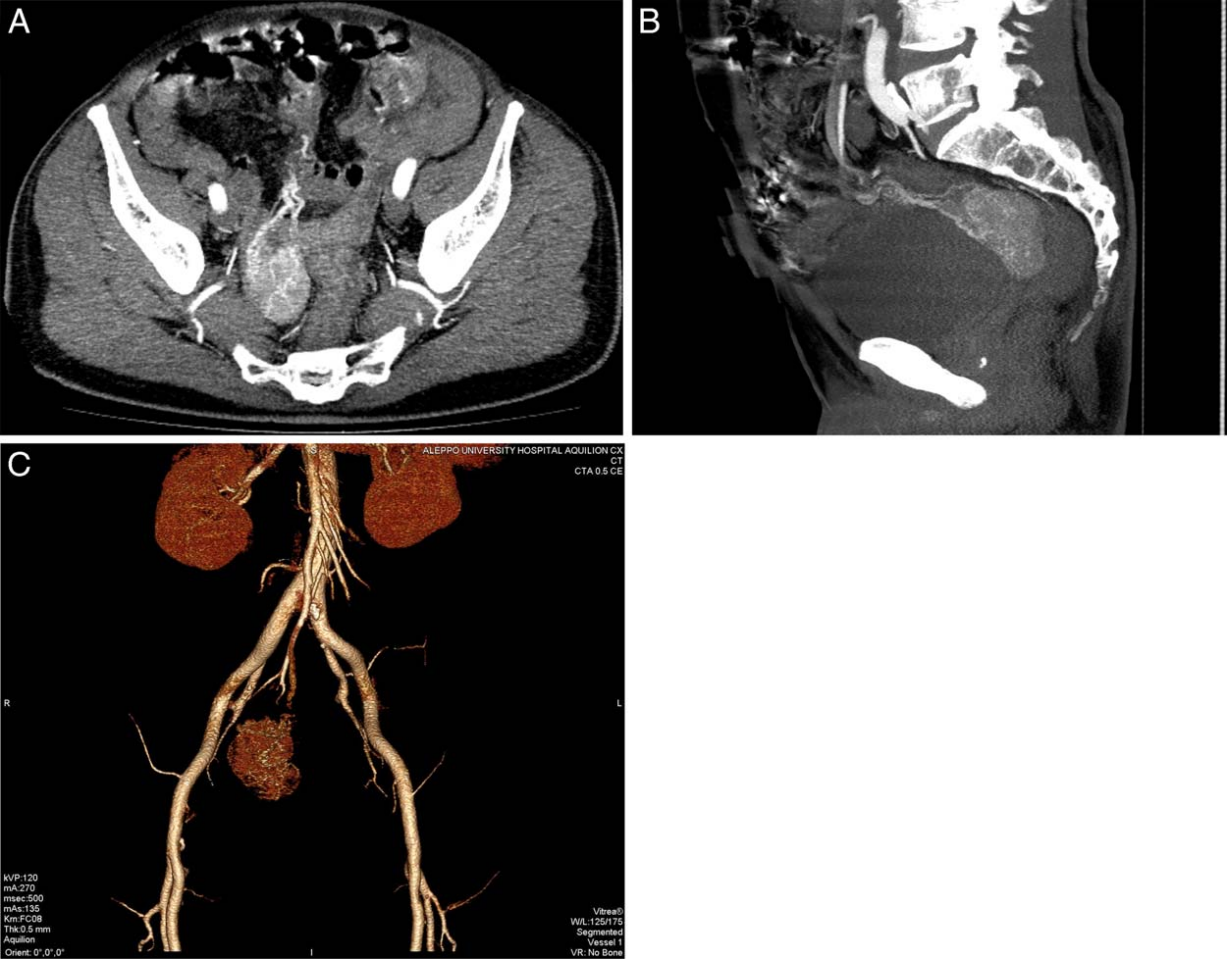
Contrast-enhanced computed tomography findings. (A, B) Axial and sagittal views in the arterial phase showcasing a well-defined hyperdense extraluminal mass in the terminal ileum situated in the retrovesical pouch. (C) Three-dimensional reconstruction of computed tomography angiography in the arterial phase showing abnormal arterial neovascularization from the superior mesenteric artery to the ileal mass.

Given the urgency of the situation, an abdominal computed tomography angiogram was ordered and revealed a well-circumscribed highly vascular lesion in the distal ileum exhibiting an exophytic component highly suggestive of a hypervascular GIST, as shown in Figure 1.

**Figure 2:**
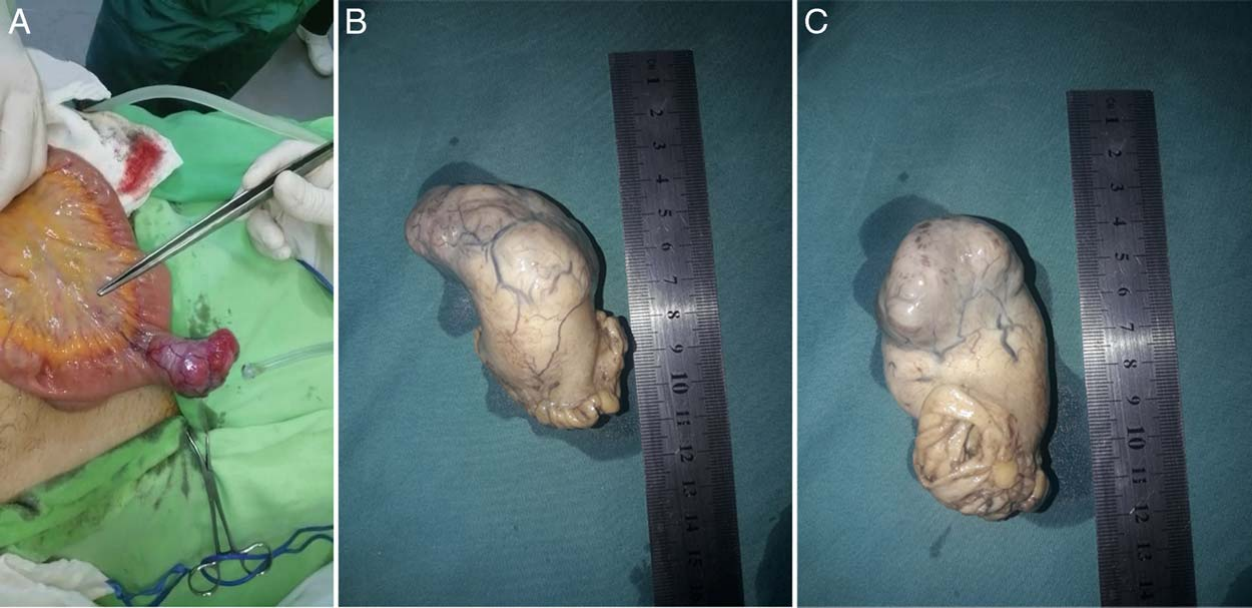
Intraoperative and postoperative findings. (A) Intraoperative view showing the hypervascular mass on the antimesenteric border of the ileum. (B, C) Gross post-resection images showing the highly vascular ileal mass.

Emergent laparotomy was performed via midline incision. Intraoperative exploration revealed the presence of an exophytic mass in the ileum measuring 5.5×4×3.5 cm as shown in Figure 2, and was about 120 cm from the duodenojejunal junction. Minimal serous fluid was noted within the intraperitoneal cavity. Damage control was performed considering the hemodynamic instability of the patient, and the ileal mass was primarily resected, ensuring its complete removal while minimizing any additional interventions that could potentially exacerbate the patient’s condition.

Following surgery, the patient was closely monitored in the ICU. After an observation period of 48 h, and after a transfusion of two units of blood, the hemoglobin levels showed signs of stabilization (9 g/dl) indicating a positive response to the treatment. The patient’s condition improved and was finally stable, warranting a transfer to the general wards. Throughout the remaining duration of the patient’s hospitalization, no complications were encountered suggesting a successful postoperative recovery with no adverse events being recorded.

Initial histopathology findings of the excised tissue were consistent with grade 1 GIST. Grossly, the specimen consisted of a brown segment of small bowel, measuring 8 cm in length and 4 cm in diameter. Attached to the antimesenteric border of the intestinal tissue was a mass, measuring 5.5×4×3.5 cm.

Microscopic analysis of sections from the mass showed diffuse proliferation of round-to-spindle tumor cells within the muscularis propria and submucosa. These tumoral cells had acidophilic cytoplasm and round to oval central nucleoli. Some of the nuclei had blunt ends, resembling the “cigar-shaped” nuclei typically observed in smooth muscle tumors, while others had sharp ends resembling those of fibroblasts. Importantly, no abnormal pathological changes were noted in the overlying mucosal layer. Immunohistochemistry studies performed on the specimen demonstrated strongly positive reactions for CD117 and DOG-1 immunostaining.

The patient was discharged on the fourth day of postoperative care. Referral to the Department of Oncology was made for further assessment and management.

## Discussion

This case highlights the challenges associated with obscure lower gastrointestinal hemorrhage and importance of computed tomography angiography in pinpointing the source of bleeding.

Previously, gastrointestinal stromal tumors were believed to have originated from smooth muscle tissue, akin to leiomyomas, leiomyosarcomas and leiomyoblastomas. However, recent research has observed mixed neural and myogenic features within these tumors, which has led to their distinct classification as GISTs^4^.

It is challenging to determine the exact origin of GISTs microscopically, but with the advent of electron microscopy and immunohistochemistry, it was suggested that these tumors originated from the interstitial cells of Cajal, which are pacemaker cells found in the intestines^1,4^.

GISTs can occur in any part of the GI tract, although they are most oftenly found in the stomach (60%), followed by the small intestines (30%), colon and rectum (5–10%), and esophagus (5%). It is noteworthy that leiomyomas account for 75% of esophageal tumors where as GISTs are more prevalent in other parts of the GI tract^4^.

As most GISTs are asymptomatic, they are not detected during a patient’s lifetime^1,4^.

The incidence of GISTs is estimated to be ~10–20 cases per million individuals each year, with a potential malignancy rate of 20–30%, and a median tumor size of 6 cm. However, the precise incidence remains uncertain due to incomplete definitions and classifications^5^.

GISTs have been reported across all age groups, including children. However, most patients between the ages of 40 and 80 at the time of diagnosis, with a median age of ~60 years. The incidence rates are similar between males and females, with a slight male predilection observed^5^.

It is important to note that the reported frequency of GISTs may change over time because of evolving diagnostic criteria and the increased awareness of these tumors among clinicians and researchers.

## Conclusion

In conclusion, this manuscript highlights the complexities and evolving understanding surrounding GISTs. The successful postoperative course of the patient serves as a valuable reminder of the importance of meticulous monitoring and prompt intervention. With the ongoing advancements in diagnostic criteria, this case contributes to our broader comprehension of these tumors, and affirms the necessity of adopting tailored treatment approaches to optimize patients’ outcomes.

## Ethical approval

This case report did not require review by the ethics committee in the Faculty of Medicine, University of Aleppo, Aleppo University Hospital (AUH), Aleppo, Syria.

## Consent

Written informed consent was obtained from the patient for publication of this case report and accompanying images. A copy of the written consent is available for review by the Editor-in-Chief of this journal on request.

## Source of funding

This research did not receive any specific grant from funding agencies in the public, commercial, or not-for-profit sectors.

## Author contribution

F.K.: contributed to data collection and interpretation, study concept and design, and writing the paper (first author and corresponding author). A.K.: contributed to data collection, study design and writing the paper (coauthor #1). T.S.: contributed to data collection and interpretation, and writing and reviewing the paper (coauthor #2). J.Z.: contributed to data collection and interpretation, study design and writing the paper (coauthor #3). D.K.: contributed to data collection and interpretation (coauthor #4). A.A.: contributed to data collection, and reviewing the paper (coauthor #5). M.M.: contributed to data collection and interpretation, and reviewing the paper (coauthor#6). N.L.: contributed to data collection and interpretation, and writing the paper (coauthor #7). Z.A.N.: contributed to data collection, and reviewing the paper (coauthor #8). A.G.: contributed to data collection, and reviewing the paper (coauthor #9).

## Conflicts of interest disclosure

The authors declare that there is no conflicts of interest.

## Research registration unique identifying number (UIN)

Registration of Research Studies: Not applicable.

Research Registration: NA.

## Guarantor

Firas Khana.

## Data availability statement

Not applicable.

## Provenance and peer review

Not commissioned; externally peer-reviewed.

## References

[1] Mahmoud S Salman H. Massive bleeding of a jejunal gastrointestinal stromal tumour: a rare case of a life-threatening presentation. J Surg Case Rep 2020;2020:rjaa355. 33062252 10.1093/jscr/rjaa355 PMC7540629

[2] Giestas S Almeida N Martins R. Small bowel GIST: clinical presentation as intussusception and obscure bleeding. GE Port J Gastroenterol 2016;23:279–281. 28868478 10.1016/j.jpge.2015.12.007 PMC5580152

[3] Sohrabi C Mathew G Maria N. The SCARE 2023 guideline: updating consensus Surgical CAse REport (SCARE) guidelines. Int J Surg Lond Engl 2023;109:1136. 10.1097/JS9.0000000000000373 PMC10389401 37013953

[4] Lee NK Kim S Kim GH. Hypervascular subepithelial gastrointestinal masses: CT-pathologic correlation. Radiographics 2010;30:1915–1934. 21057127 10.1148/rg.307105028

[5] Daldoul S Moussi A Triki W. Jejunal GIST causing acute massive gastrointestinal bleeding: Role of multidetector row helical CT in the preoperative diagnosis and management. Arab J Gastroenterol 2012;13:153–157. 23122460 10.1016/j.ajg.2012.08.005

